# Association of exercise blood pressure relative to fitness with coronary artery disease

**DOI:** 10.1136/openhrt-2026-004514

**Published:** 2026-10-21

**Authors:** Masafumi Takafuji, Hafiz Naderi, Ching-Hui Sia, Bradley Chambers, Maryum Farooq, Thomas Anderton, Sven Plein, John P Greenwood, Peter P Swoboda

**Affiliations:** 1 Leeds Institute of Cardiovascular and Metabolic Medicine, University of Leeds, Leeds, UK; 2 Department of Radiology, Mie University Hospital, Tsu, Japan; 3 William Harvey Research Institute, Queen Mary University of London, London, UK; 4 Barts Heart Centre, St Bartholomew’s Hospital, London, UK; 5 Baker Heart and Diabetes Institute and University of Melbourne, Melbourne, Victoria, Australia

**Keywords:** CORONARY ARTERY DISEASE, RISK STRATIFICATION, Hypertension, Coronary Angiography, Magnetic Resonance Imaging

## Abstract

**Background:**

Exercise systolic blood pressure (SBP) indexed to fitness, expressed as SBP divided by peak metabolic equivalents (SBP/MET_peak_), is associated with cardiovascular events in patients undergoing exercise testing. We aimed to characterise the cardiac phenotype and outcomes of patients with elevated exercise SBP indexed to fitness.

**Methods:**

We analysed 530 participants with suspected angina from the CE-MARC trial who had complete exercise treadmill testing, invasive coronary angiography (ICA) and cardiovascular magnetic resonance (CMR) imaging. High SBP/MET_peak_ was defined as ≥18 mm Hg/METs. Obstructive coronary artery disease (CAD) was defined by quantitative ICA as ≥70% stenosis in a major epicardial artery. Inducible ischaemia and myocardial scar were assessed by stress perfusion and late gadolinium enhancement CMR, respectively. Major adverse cardiovascular events (MACE) were defined as all-cause death and acute coronary syndrome.

**Results:**

High SBP/MET_peak_ was present in 310 participants (58.5%) and prevalence increased progressively with the number of diseased vessels, presence of ischaemia and myocardial scar (p<0.01 for all). During a median follow-up of 15.9 years, high SBP/MET_peak_ was associated with MACE after adjustment for age, sex, obstructive CAD, ischaemia and myocardial scar (adjusted HR per 1-unit increase, 1.02 (95% CI 1.00 to 1.03), p=0.012). Higher SBP/MET_peak_ identified a worse prognosis in subgroups with and without obstructive CAD and in those with and without ischaemia or infarction (log-rank, p<0.005 for all).

**Conclusions:**

Among patients with suspected angina, high SBP/MET_peak_ was associated with myocardial ischaemia and scar and adverse long-term outcomes. However, this association appeared to be driven predominantly by lower exercise capacity rather than peak SBP. SBP/MET_peak_ may therefore represent an exploratory integrated exercise marker requiring external validation.

**WHAT IS ALREADY KNOWN ON THIS TOPIC**

- Exercise systolic blood pressure (SBP) is associated with cardiovascular risk, but its interpretation is strongly influenced by achieved workload and fitness; indexing SBP to peak metabolic equivalents (SBP/MET_peak_) has been associated with cardiovascular events, although its relationship with objective coronary and myocardial abnormalities was unknown.

**WHAT THIS STUDY ADDS**

- In patients with suspected angina, higher SBP/MET_peak_ was associated with greater obstructive coronary artery disease burden, cardiovascular magnetic resonance-defined inducible ischaemia, myocardial scar and adverse long-term outcomes. Although SBP/MET_peak_ remained associated with outcome after multivariable adjustment and improved overall model fit, its incremental contribution to risk discrimination was small.

**HOW THIS STUDY MIGHT AFFECT RESEARCH, PRACTICE OR POLICY**

- Because SBP/MET_peak_ can be derived from routinely collected exercise-test variables, it may provide a simple complementary marker for cardiovascular risk stratification. However, because its prognostic information appears to be driven largely by exercise capacity and its incremental discriminatory value was modest, external validation is required before routine clinical application.

## Introduction

 Blood pressure (BP) measured during exercise stress testing provides an integrated assessment of cardiovascular performance under physiological demand.^1^ Although exercise testing is primarily used to evaluate symptoms, functional capacity, arrhythmia and inducible myocardial ischaemia, BP is routinely recorded and may contain additional prognostic information.^2^ A hypertensive response to exercise has been associated with future hypertension, cardiovascular events and mortality, independent of resting BP and conventional cardiovascular risk factors, and may identify a phenotype characterised by masked or uncontrolled hypertension, arterial stiffness, impaired vasodilator reserve, sympathetic activation and hypertension-mediated organ damage.^3^

However, the interpretation of exercise systolic BP (SBP) is challenging. Peak SBP is strongly influenced by achieved workload and cardiorespiratory fitness.^4^ A high peak SBP may be physiological in a fit individual exercising to a high workload, whereas a similar or lower pressure at low exercise capacity may indicate abnormal vascular loading and higher cardiovascular risk.^5^ This has contributed to inconsistent thresholds for exaggerated exercise BP response and limited implementation. Exercise SBP indexed to achieved fitness, expressed as SBP divided by peak metabolic equivalents (SBP/MET_peak_) has recently been proposed as a simple clinically available metric. In the EXERTION study, SBP/MET_peak_ was strongly associated with fatal and nonfatal cardiovascular events, whereas exercise SBP without consideration of fitness was not associated with outcomes.^4^ However, it is currently unknown whether this BP–fitness phenotype is associated with objective markers of coronary atherosclerosis and myocardial infarction. This is clinically relevant as patients referred for investigation of suspected angina often have overlapping mechanisms of risk, including resting hypertension, impaired exercise capacity, obstructive coronary artery disease (CAD), inducible ischaemia and unrecognised myocardial infarction. The CE-MARC study provides an ideal setting to examine this question as participants underwent invasive coronary angiography (ICA), cardiovascular magnetic resonance (CMR), single-photon emission CT (SPECT) and in most patients exercise tolerance testing, with long-term outcome follow-up.^6–12^ CMR further enables detection of inducible ischaemia and myocardial scar by late gadolinium enhancement (LGE), both of established diagnostic and prognostic relevance.^9 13 14^

We therefore investigated the association of exercise SBP relative to fitness with obstructive CAD, CMR-defined inducible ischaemia, myocardial scar and clinical outcomes in patients from CE-MARC with suspected angina.

## Methods

### Design

The design and primary outcome of CE-MARC have been previously published.^6–12^ CE-MARC studied patients referred to cardiologists for suspected angina pectoris. All patients then underwent SPECT and CMR, randomised to test order, followed by ICA, regardless of the treating cardiologist’s clinical management intention or prior test results. Exercise tolerance testing (ETT) was performed if patients were physically able.

Patients and the public were not involved in the design or conduct of this retrospective observational study, the interpretation of the results or the preparation or dissemination of the manuscript.

### Patients

Patients were recruited between March 2006 and August 2009 from two UK secondary and tertiary care hospitals. Consecutive patients were screened and enrolled if they had at least one major cardiovascular risk factor and a cardiologist considered them to have possible stable angina requiring further cardiac investigation in accordance with contemporary clinical practice at the time of the trial. Inclusion for this analysis included all CE-MARC patients who underwent all of CMR, ICA and ETT. Exclusion criteria were previous coronary artery bypass surgery, crescendo angina or acute coronary syndrome, contraindication to CMR (eg, pacemaker) or adenosine infusion (reversible airways disease, atrioventricular heart block), pregnancy, inability to lie supine, estimated glomerular filtration rate ≤30 mL/min/1.73m^2^ and subjects without records of BP and METs during ETT.

Among the final study population, patients who underwent SPECT were analysed as a subgroup to assess the incremental value of SBP/MET beyond SPECT.

### Investigational procedures and their analysis

Detailed procedural protocols and results have been published.^6–12^ A brief description is given below.

### Exercise tolerance testing

ETT was performed with treadmill testing using a Bruce or modified Bruce protocol. All exercise ECGs were supervised and reviewed by experienced technical staff. The CE-MARC research database contained resting heart rate and BP, maximum achieved heart rate and BP, exercise duration, symptoms, reasons for test termination and MET_peak_ calculated by the exercise-testing software. One SBP/MET_peak_ value was calculated for each participant as maximum achieved exercise SBP divided by peak METs and was expressed as mm Hg/METs. A value of 18 mm Hg/METs was evaluated as a previously proposed pragmatic threshold rather than as a universally validated optimal cut-off.^4^

### Cardiovascular magnetic resonance

CMR was performed on a 1.5 T Philips Intera system (Philips, Best, The Netherlands) using a protocol that included stress perfusion (adenosine, 140 µg/kg/min for ≥4 min), cine imaging, rest perfusion and LGE. Perfusion CMR and LGE CMR scans were analysed in a blinded manner by paired readers with >10 years’ experience and this analysis was based on the original trial read.^6–12^ Specific imaging and reporting parameters for CMR have been described previously.^7^ For this analysis, a positive CMR result was defined as the presence of either myocardial ischaemia or myocardial scar.

### X-ray angiography

All X-ray angiograms were performed after CMR and SPECT and reported by two experienced interventional cardiologists blinded to the other studies. Obstructive CAD was defined as ≥70% stenosis of a first-order coronary artery measuring ≥2 mm in diameter or left main stem stenosis ≥50%, by quantitative coronary angiography (QCA) using QCA Plus software (Sanders Data Systems, Palo Alto, California, USA), with a post-stenosis diameter used as the reference vessel diameter in cases of ostial disease.^6 7 9^

### Single-photon emission CT

SPECT was performed using a 2-day adenosine stress/rest gated 99mTc-tetrofosmin protocol. Stress imaging was acquired during intravenous adenosine infusion (140 µg/kg/min for 4 min) followed by tracer injection.^6–12^

#### Follow-up

Follow-up was completed by research nurses blinded to study data, who had access to electronic health records for consented patients. The median follow-up duration was 15.9 years (IQR 14.8–16.8 years). A detailed medical history since randomisation was obtained from all hospital and general practitioners’ records and cross-referenced to information obtained by direct telephone contact with each patient. The primary endpoint was major adverse cardiovascular events (MACE), defined as the composite of all-cause death or acute coronary syndrome. Secondary exploratory endpoints were acute coronary syndrome, all-cause death, cardiovascular death and non-cardiovascular death.

#### Statistics

Statistical analyses were performed using JMP V.14 software (SAS Institute, Cary, North Carolina, USA). For variables with missing values, no imputation was performed; analyses were conducted using the available data for each variable. Continuous variables are expressed as median (IQR). Comparisons among quantitative variables were performed using the independent-samples Mann-Whitney U test. Categorical data were compared using Pearson χ^2^ tests.

Separate multivariable logistic regression models adjusted for age and sex were used to evaluate associations of SBP/MET_peak_ with the binary outcomes of obstructive CAD, inducible ischaemia and myocardial scar.

Kaplan-Meier curves were used to estimate survival probabilities and compare time the event rate of MACE and all-cause mortality in patients with and without high SBP/MET. The log-rank test was used to compare survival curves.

Cox proportional-hazards regression was used for time-to-event analyses. Component-based models separately evaluated peak METs, peak SBP, both variables entered together and SBP/MET_peak_; these models were adjusted for age, sex, obstructive CAD, inducible ischaemia and myocardial scar. A clinical model additionally adjusted for age, sex, body mass index (BMI), hypertension, diabetes, current smoking, resting SBP and baseline use of ACE inhibitors, beta-blockers and calcium-channel blockers. Endpoint-specific exploratory models were adjusted for age and myocardial scar. A sensitivity analysis excluded participants who underwent a modified Bruce protocol.

Incremental prognostic value was evaluated by comparing nested models with and without continuous SBP/MET_peak_ using likelihood-ratio tests, corrected Akaike information criterion (AICc), Harrell’s C-statistic, time-dependent area under the receiver operating characteristic (ROC) curve (AUC). Time-dependent ROC analyses were performed at 5, 10 and 15 years, with 15 years designated as the primary horizon. The exploratory 15-year cut-off was selected by maximising the time-dependent Youden index. The proportional-hazards assumption was assessed using Schoenfeld residuals. Non-linearity was examined using a restricted cubic spline Cox model with knots at the 10th, 50th and 90th percentiles and 18 mm Hg/METs as the reference, adjusted for age, sex, CAD, ischaemia and myocardial scar. All tests were deemed to be statistically significant when p<0.05.

## Results

Of 752 patients randomised in the CE-MARC trial, 539 patients had complete data for ETT, CMR and ICA. 530 patients remained after excluding nine patients with missing maximal SBP or METs results. High SBP/MET_peak_ occurred in 310/530 (58.5%) participants. Among 530 participants, 518 (97.7%) underwent the standard Bruce protocol and 12 (2.3%) underwent the modified Bruce protocol. The prevalence of obstructive CAD at invasive angiography in the population was 38.7% (205/530), and myocardial scar at LGE-CMR was seen in 16.2% (86/530), which was comparable to the prevalences reported for the whole CE-MARC trial population.^7^ Baseline patient characteristics in relation to SBP/MET_peak_ are summarised in table 1. Participants with higher SBP/MET_peak_ were older, less frequently male and had higher BMI, with a greater prevalence of hypertension, diabetes and use of ACE inhibitors, statins and diuretics, but a lower prevalence of current smoking. In terms of left ventricular (LV) volumetric parameters, higher SBP/MET_peak_ was associated with smaller LV end-diastolic volume index (LVEDVi) and a higher LV mass-to-volume ratio (table 2) but no difference in LV mass index or LV ejection fraction.

**Table 1:** Baseline patient demographics in relation to exercise systolic blood pressure relative to fitness

Variable median (IQR) or n (%)	All patients (n=530)	SBP/MET_peak_ ≥18_ mm Hg/METs_ (n=310)	SBP/MET_peak_ <18 _mm Hg/METs_ (n=220)	P value
Patient background				
Age (years)	59 (52–67)	63 (56–69)	56 (48–63)	<0.001
Male	335 (63)	181 (58)	154 (70)	0.006
BMI (kg/m^2^)	28.7 (25.7–31.2)	29.2 (26.0–32.0)	27.7 (25.3–30.4)	<0.001
Hypertension	259 (49)	175 (56)	84 (38)	<0.001
Diabetes	53 (10)	42 (14)	11 (5)	0.001
Total cholesterol (mmol/L)	5.2 (4.4–6.1)	5.2 (4.3–6.0)	5.3 (4.6–6.1)	0.173
Current smoking	88 (17)	43 (14)	45 (20)	0.045
Past smoking	248 (47)	150 (48)	98 (45)	0.383
Beta blocker	148 (28)	83 (27)	65 (30)	0.484
ACEi	159 (30)	110 (35)	49 (22)	0.001
Calcium channel blocker	70 (13)	49 (16)	21 (10)	0.036
Nitrate	220 (42)	137 (44)	83 (38)	0.137
Diuretic	69 (13)	58 (19)	11 (5)	<0.001
Aspirin	293 (55)	175 (56)	118 (54)	0.521
Statin	205 (39)	140 (45)	65 (30)	<0.001
History of ACS	32 (6)	18 (6)	14 (6)	0.791
Clinical outcome				
Composite of death and ACS	142 (27)	111 (36)	31 (14)	<0.001
Death	117 (22)	97 (31)	20 (9)	<0.001
Cardiovascular death	22 (4)	19 (6)	3 (1)	0.007
Non-cardiovascular death	95 (18)	78 (25)	17 (8)	<0.001
ACS	28 (5)	16 (5)	12 (5)	0.882

ACEi, ACE inhibitor; ACS, acute coronary syndrome; BMI, body mass index; SBP/MET_peak_, systolic blood pressure and peak metabolic equivalents.

**Table 2:** Exercise tolerance test, invasive coronary angiography and CMR parameters of the patients in relation to exercise systolic BP relative to fitness

Variable median (IQR) or n (%)	All patients (n=530)	SBP/MET_peak_ ≥18_mm Hg/METs_ (n=310)	SBP/MET_peak_ <18_mm Hg/METs_ (n=220)	P value
Exercise tolerance test				
Rest systolic BP (mm Hg)	136 (120–150)	140 (128–152)	128 (115–140)	<0.001
Rest diastolic BP (mm Hg)	80 (70–84)	80 (70–86)	78 (70–82)	0.003
Rest HR (bpm)	76 (65–85)	77 (67–87)	74 (63–83)	0.002
Maximal systolic BP (mm Hg)	175 (160–194)	180 (160–200)	170 (154–180)	<0.001
Maximal diastolic BP (mm Hg)	80 (70–86)	80 (70–90)	80 (70–84)	<0.001
Maximal HR (bpm)	145 (128–161)	141 (125–155)	154 (136–168)	<0.001
Exercise duration (min)	6.4 (4.5–8.9)	4.8 (3.5–6.2)	9.2 (7.2–10.1)	<0.001
METs	10.2 (7.0–10.2)	7.0 (7.0–7.0)	11.7 (10.2–13.5)	<0.001
EIH	107 (20)	86 (28)	21 (10)	<0.001
Chest pain during ETT	96 (18)	65 (21)	31 (14)	0.043
ECG abnormality during ETT	66 (12)	42 (14)	24 (11)	0.365
Protocol				0.003
Bruce protocol	518 (98)	298 (96)	220 (100)	
Modified Bruce	12 (2)	12 (4)	0 (0)	
Invasive coronary angiography				
Obstructive CAD	205 (39)	140 (45)	65 (30)	<0.001
Number of VD				<0.001
Non-obstructive CAD	325 (61)	170 (55)	155 (70)	
One vessel-disease	107 (20)	68 (22)	39 (18)	
Two vessel-disease	64 (12)	43 (14)	21 (10)	
Three vessel-disease	34 (6)	29 (9)	5 (2)	
CMR parameters				
LV EDVi (mL/m^2^)	78.4 (69.8–86.9)	76.4 (68.3–85.2)	81.1 (72.7–90.5)	<0.001
LVEF (%)	56.5 (53.0–60.3)	56.8 (53.1–60.4)	56.3 (53.0–60.1)	0.659
LV mass (g/m^2^)	47.4 (42.1–53.9)	47.3 (42.1–53.5)	48.1 (42.6–54.6)	0.446
LV mass/volume ratio (g/mL)	0.61 (0.54–0.68)	0.62 (0.55–0.69)	0.59 (0.53–0.65)	0.003
Inducible ischaemia	218 (41)	148 (48)	70 (32)	<0.001
Ischaemia score if present	3 (2–5)	4 (2–6)	3 (1–5)	0.030
Myocardial scar	86 (16)	62 (20)	24 (11)	0.005
Scar mass (g) if present	4.9 (2.3–11.2)	4.7 (2.3–11.0)	6.5 (2.0–12.6)	0.989

BP, blood pressure; CAD, coronary artery disease; CMR, cardiovascular magnetic resonance; EDVi, end-diastolic volume index; EIH, exercise-induced hypertension; ETT, exercise tolerance testing; HR, heart rate; ICA, invasive coronary angiography; LV, left ventricular; LVEF, left ventricular ejection fraction; SBP/MET_peak_, systolic blood pressure and peak metabolic equivalents.

Among the 530 patients, 512 underwent SPECT and 34.2% (175/512) showed positive findings on SPECT.

### Association of SBP/MET_peak_ and CAD

The prevalence of high SBP/MET_peak_ was lowest in those without obstructive CAD and increased progressively with greater angiographic disease extent: no obstructive CAD 52.3% (170/325), one-vessel 63.6% (68/107), two-vessel 67.2% (43/64), three-vessel 85.3% (29/34) (p<0.001). Participants with inducible ischaemia had a significantly higher frequency of high SBP/MET_peak_ than those without inducible ischaemia (67.9% (148/218) vs 51.9% (162/310) p<0.001). Similarly, participants with myocardial scar also had a significantly higher frequency of high SBP/MET_peak_ than those without scar (72.1% (62/86) vs 55.9% (248/444), p<0.001) (table 2, online supplemental figure 1).

In separate logistic models adjusted for age and sex, higher SBP/MET_peak_ was associated with CAD (OR 1.02 per 1 mm Hg/METs, 95% CI 1.00 to 1.05; p=0.021), inducible ischaemia (OR 1.03, 95% CI 1.01 to 1.05; p=0.007) and myocardial scar (OR 1.04, 95% CI 1.02 to 1.06; p<0.001).

Among patients without a history of ACS but myocardial scar detected by LGE-CMR, SBP/MET_peak_>18 mm Hg/METs was more common than <18 mm Hg/METs, but this did not reach significance (77% vs 23%, OR 2.45, 95% CI 0.90 to 6.69, p=0.081) (online supplemental table 1).

### Prognostic value of SBP/MET_peak_

During follow-up, 142 participants experienced MACE, including 111 in the SBP/MET_peak_≥18 mm Hg/METs group and 31 in the SBP/MET_peak_<18 mm Hg/METs group. There were 28 acute coronary syndrome events and 117 deaths, including 22 cardiovascular deaths and 95 non-cardiovascular deaths. Kaplan-Meier curve analysis showed that higher SBP/MET_peak_ was associated with MACE and all-cause death (MACE, p<0.001; all-cause death, p<0.001) (figure 1). In Cox proportional hazards analysis, higher SBP/MET_peak_ was associated with MACE in univariate analysis (HR per 1-unit increase, 1.03 (95% CI 1.02 to 1.04), p<0.001), and multivariate analysis after adjustment for age, sex, presence of obstructive CAD, inducible ischaemia and myocardial scar (HR per 1-unit increase 1.02 (95% CI 1.00 to 1.03), p=0.012) (table 3). Age (HR per 1-unit increase, 1.08 (95% CI 1.06 to 1.10), p<0.001) and myocardial scar (HR 1.71 (95% CI 1.09 to 2.69), p=0.020) were significantly associated with MACE. In the restricted cubic spline model, the overall association was significant (p=0.010), with evidence of non-linearity (p for non-linearity=0.020). Adjusted hazard increased with SBP/MET_peak_, with attenuation of the increase at higher values (online supplemental figure 2). In exploratory Cox models adjusted for age and myocardial scar, SBP/MET_peak_ was associated with all-cause mortality and cardiovascular death, but no statistically significant association was detected with non-cardiovascular death or acute coronary syndrome (online supplemental table 2). There was no evidence of violation of the proportional-hazards assumption for SBP/MET_peak_ (p=0.404) or globally (p=0.395; online supplemental table 3).

**Figure 1:**
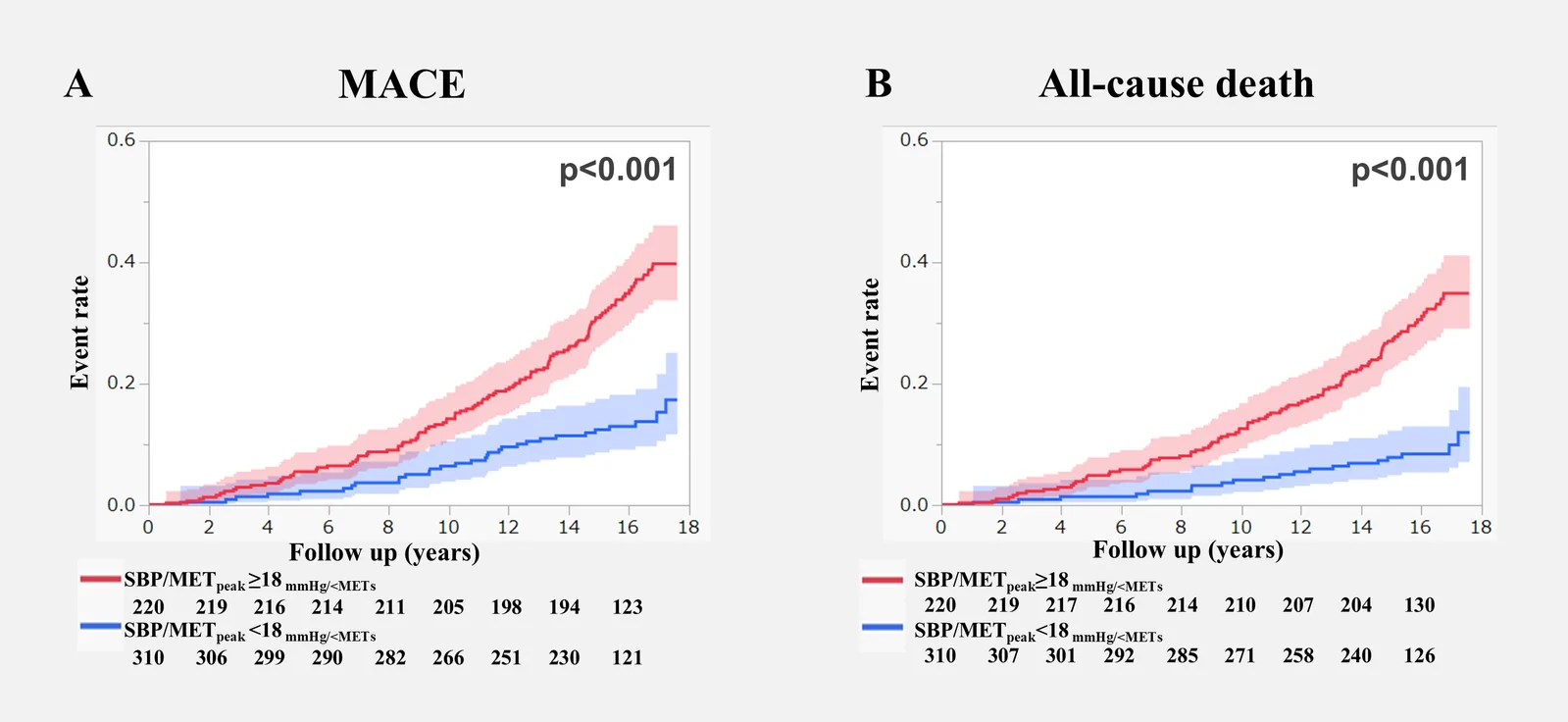
Kaplan-Meier survival curves according to systolic blood pressure relative to fitness for MACE and all-cause mortality. Kaplan-Meier curve analysis showed that SBP/MET_peak_ was associated with MACE (**A**) and all-cause mortality (**B**). MACE, major adverse cardiovascular events; SBP/MET_peak_, systolic blood pressure and peak metabolic equivalents.

**Table 3:** Univariable and multivariable Cox proportional hazard analysis of exercise systolic blood pressure relative to fitness for predicting major adverse cardiovascular events

	Univariable analysis		Multivariable analysis	
**Predictor**	**HR (95% CI**)	**P value**	**HR (95% CI**)	**P value**
SBP/MET_peak_	1.03 (1.02 to 1.04)	<0.001	1.02 (1.00 to 1.03)	0.012
Age	1.09 (1.07 to 1.11)	<0.001	1.08 (1.06 to 1.10)	<0.001
Male	1.31 (0.92 to 1.86)	0.131	1.18 (0.80 to 1.72)	0.402
Obstructive CAD	1.80 (1.30 to 2.51)	<0.001	1.05 (0.65 to 1.71)	0.842
Inducible ischaemia on CMR	1.52 (1.10 to 2.12)	0.012	0.99 (0.62 to 1.58)	0.973
Myocardial scar on CMR	2.25 (1.55 to 3.26)	<0.001	1.71 (1.09 to 2.69)	0.020

HRs (95% CIs) were expressed per 1-unit increase in SBP/MET_peak_ and age.

CAD, coronary artery disease; CMR, cardiovascular magnetic resonance; SBP/MET_peak_, systolic blood pressure and peak metabolic equivalents.

The time-dependent AUC of continuous SBP/MET_peak_ was 0.670 at 5 years, 0.623 at 10 years and 0.669 (95% CI 0.613 to 0.725) at 15 years. At 15 years, the previously proposed cut-off of 18 mm Hg/METs had sensitivity 77.9% and specificity 47.0%. The exploratory cut-off maximising the 15-year time-dependent Youden index was 20.6 mm Hg/METs, with sensitivity 71.4%, specificity 60.8% and Youden index 0.322 (online supplemental table 4).

In the clinical model adjusted for age, sex, BMI, hypertension, diabetes, current smoking, resting SBP and recorded antihypertensive medications, SBP/MET_peak_ remained associated with the primary outcome (adjusted HR 1.02, 95% CI 1.01 to 1.03; p<0.001). In a sensitivity analysis restricted to the 518 participants who underwent the standard Bruce protocol, the association was similar (adjusted HR 1.02, 95% CI 1.00 to 1.03; p=0.018).

In models adjusted for age, sex, obstructive CAD, inducible ischaemia and myocardial scar, peak METs were associated with the primary outcome when entered alone (HR 0.88, 95% CI 0.82 to 0.94; p<0.001), whereas peak SBP was not (HR 1.00, 95% CI 0.99 to 1.00; p=0.145). When peak METs and peak SBP were entered together, peak METs remained associated with the outcome (HR 0.88, 95% CI 0.83 to 0.94; p<0.001), whereas peak SBP remained non-significant (HR 1.00, 95% CI 0.99 to 1.00; p=0.321). SBP/MET_peak_ was associated with the primary outcome in the corresponding model (HR 1.02, 95% CI 1.00 to 1.03; p=0.012), but the peak-METs model had better model fit (AICc=1626; global χ²=101.2) than the SBP/MET_peak_ model (AICc=1636; global χ²=90.9) (online supplemental table 5).

#### Incremental prognostic value of SBP/MET_peak_ beyond X-ray angiography, CMR or SPECT

When patients were categorised into four subgroups according to the presence or absence of higher SBP/MET_peak_ and obstructive CAD by ICA, or inducible ischaemia and/or myocardial scar by CMR, Kaplan-Meier curve analysis demonstrated that patients with both higher SBP/MET_peak_ and obstructive CAD or inducible ischaemia/myocardial scar had the worst prognosis, whereas those with neither had the most favourable prognosis (figure 2). Higher SBP/MET_peak_ identified a worse prognosis in subgroups with and without obstructive CAD (p<0.005 for both) and in those with and without ischaemia and/or infarction (p<0.005 for all). A similar pattern was observed when patients were categorised into four subgroups according to the presence or absence of higher SBP/MET_peak_ and abnormal SPECT (figure 2).

**Figure 2:**
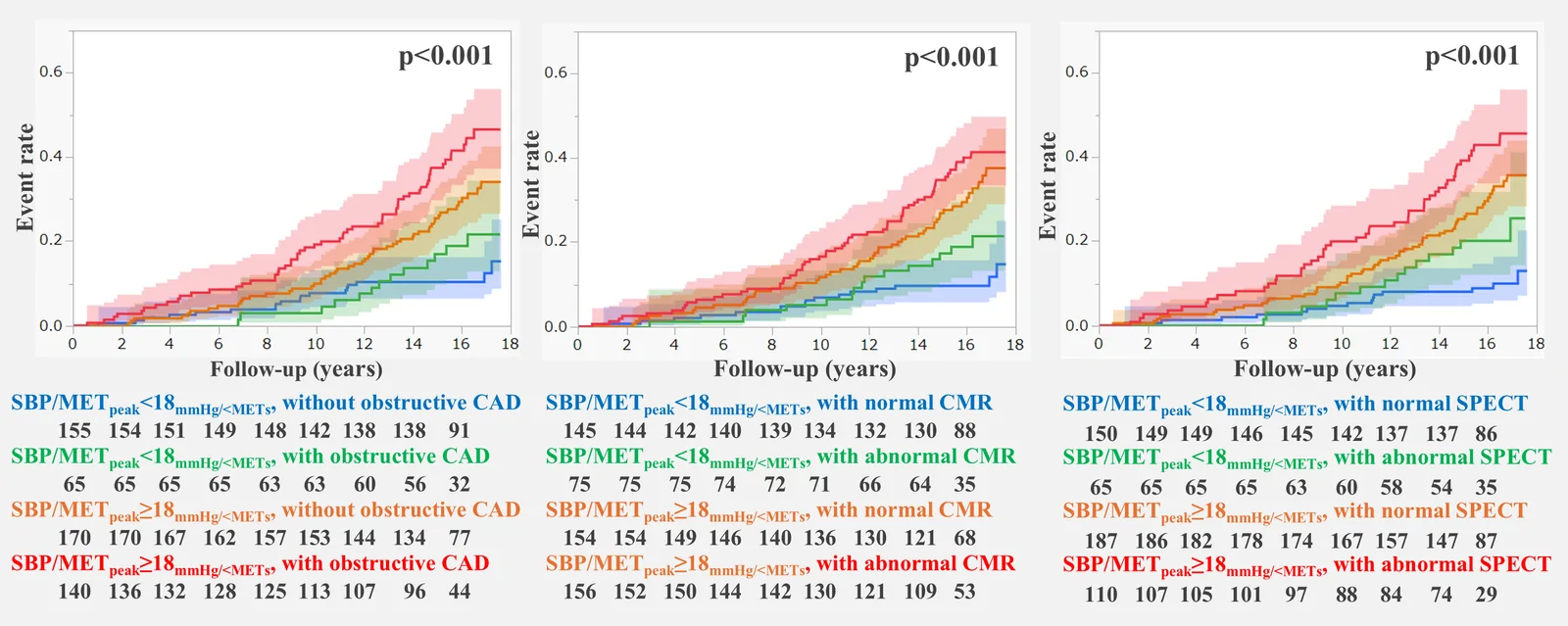
Incremental prognostic value of systolic blood pressure relative to fitness beyond invasive coronary angiography, CMR or SPECT Kaplan-Meier curves showing event rate according to the presence or absence of higher SBP/MET_peak_ combined with obstructive coronary artery disease, inducible ischaemia/myocardial scar by CMR or abnormal SPECT findings. CAD, coronary artery disease; CMR, cardiovascular magnetic resonance; SBP/MET_peak_, systolic blood pressure and peak metabolic equivalents; SPECT, single-photon emission CT.

Adding SBP/MET_peak_ improved overall model fit by likelihood-ratio testing for the clinical plus CAD model (χ²=7.04; p=0.008), clinical plus CMR abnormality model (χ²=7.16; p=0.007) and clinical plus SPECT abnormality model (χ²=7.80; p=0.005). However, increases in Harrell’s C-statistic were small (0.006–0.007). At 15 years, changes in time-dependent AUC were also small and non-significant for the CAD, CMR and SPECT models (all p≥0.511) and were essentially unchanged (online supplemental table 6).

## Discussion

In this secondary analysis of the CE-MARC trial, exercise SBP relative to achieved fitness was associated with coronary atherosclerotic burden, CMR-defined myocardial ischaemia, myocardial scar, and long-term clinical outcomes in patients investigated for suspected angina. High SBP/MET_peak_ was common, occurring in more than half of the study population, and was associated with an adverse clinical risk profile, including older age, higher BMI, hypertension, diabetes and greater use of cardiometabolic medications. Higher SBP/MET_peak_ was also associated with smaller LVEDVi and a higher LV mass-to-volume ratio, without higher LV mass index, consistent with a more concentric pattern of left ventricular remodelling. SBP/MET_peak_ remained associated with outcome after adjustment for clinical and imaging variables, but its incremental improvement in discrimination and prediction error was small.

These findings extend the clinical relevance of exercise BP relative to fitness from population-level cardiovascular risk prediction to clinically relevant disease phenotyping in suspected CAD. The EXERTION study demonstrated that SBP/MET_peak_ was associated with fatal and nonfatal cardiovascular events in a large multicentre cohort undergoing treadmill exercise testing, whereas exercise SBP considered without fitness was not associated with outcomes.^4^ Our findings extend those observations by showing that SBP/MET_peak_ is also associated with objective markers of obstructive CAD, inducible ischaemia, myocardial scar and prognosis in a deeply phenotyped cohort.

Exercise SBP rises physiologically during dynamic exercise as cardiac output increases, so interpretation requires consideration of achieved workload and cardiorespiratory fitness.^2^ Conventional definitions of exaggerated exercise BP response based on absolute peak SBP thresholds do not fully account for this distinction.^15^ Prior studies have suggested that BP measured during moderate or submaximal exercise may be more consistently associated with future cardiovascular events than peak SBP alone,^16 17^ supporting fitness-indexed exercise BP measures. A high SBP/MET_peak_ may reflect higher exercise SBP, lower achieved exercise capacity, or both, thereby capturing the pressure generated for a given level of metabolic work. Higher exercise SBP may reflect arterial stiffness, impaired endothelial vasodilator reserve, increased sympathetic activation, masked or uncontrolled hypertension and adverse cardiometabolic risk.^16 18^ Lower achieved METs, in turn, may reflect impaired cardiovascular reserve, deconditioning, obesity, pulmonary limitation, ischaemia or comorbidity.^3^ In the current study, the component-based analyses, in which peak METs remained prognostic but peak SBP did not, indicate that the observed prognostic association was driven predominantly by exercise capacity.

The association between SBP/MET_peak_ and angiographic CAD is clinically important. The prevalence of high SBP/MET_peak_ increased progressively with angiographic disease extent, from patients without obstructive CAD to those with three-vessel disease. Higher SBP/MET_peak_ was also associated with inducible ischaemia on CMR. Potential mechanisms include cumulative vascular risk from hypertension, diabetes, obesity and smoking; increased myocardial oxygen demand from a disproportionate pressure response at low workload; and arterial stiffness and impaired vasodilator reserve coexisting with coronary microvascular dysfunction, further worsening myocardial oxygen supply-demand mismatch.^19^ Conversely, established CAD and ischaemia may reduce exercise capacity, increasing SBP/MET_peak_ through the denominator. Therefore, SBP/MET_peak_ should be interpreted as an integrated exercise phenotype rather than as a pure measure of vascular function.

LGE by CMR identifies irreversible myocardial injury and has established prognostic importance.^13 20^ In this study, both myocardial scar and SBP/MET_peak_ were independently associated with the primary outcome, suggesting complementary information; myocardial scar reflects accumulated structural injury, whereas SBP/MET_peak_ reflects the haemodynamic and functional response to exercise. Myocardial scar without a clinical history of acute coronary syndrome tended to be more frequent in patients with high SBP/MET_peak_ although this association did not reach statistical significance.

In the current study, patients with both high SBP/MET_peak_ and obstructive CAD, abnormal CMR or abnormal SPECT had the worst outcomes, whereas those with low SBP/MET_peak_ and no abnormal imaging had the most favourable prognosis. High SBP/MET_peak_ identified higher-risk patients even among those without obstructive CAD, without abnormal CMR or SPECT. Furthermore, adding SBP/MET_peak_ improved likelihood-based model fit. However, changes in Harrell’s C-statistic and 15-year time-dependent AUC were small. The four-group Kaplan-Meier analyses should therefore be interpreted as descriptive risk separation rather than proof of clinically meaningful incremental prediction.

This study has several strengths. CE-MARC included a prospective, well-characterised cohort of patients with suspected angina who underwent ICA, ETT, SPECT and multiparametric CMR, with long-term follow-up. This comprehensive assessment allowed SBP/MET_peak_ to be evaluated in relation to coronary anatomy, ischaemia, myocardial scar and clinical outcomes. Blinded imaging interpretation further reduced potential assessment bias.

However, several limitations of this trial post hoc analysis should be acknowledged. This post hoc analysis included only patients with suspected angina who were able to exercise, which may limit generalisability to other populations and contemporary diagnostic pathways. METs were estimated from treadmill workload rather than measured directly, and the concentration at 7.0 METs partly reflected stage-based estimation in the Bruce protocol. Stage-specific SBP measurements were unavailable, precluding stage-specific SBP/MET_peak_ analyses. Only 12 participants underwent the modified Bruce protocol, although the Bruce-only sensitivity analysis was consistent with the main result. The 20.6 mm Hg/METs cut-off was derived in the same cohort and may be optimistic. Endpoint-specific and spline analyses were exploratory, and the observational design does not establish causality. External validation is required.

## Conclusion

In CE-MARC participants with suspected angina, higher maximum exercise SBP relative to peak METs was associated with CAD, inducible ischaemia, myocardial scar and long-term outcomes. Component analyses indicated that the prognostic association predominantly reflected lower exercise capacity rather than peak SBP. Although SBP/MET_peak_ remained associated with outcome after multivariable adjustment and improved overall model fit, its incremental contribution to discrimination was small. It should therefore be regarded as an exploratory integrated exercise marker requiring external validation, rather than an independently established haemodynamic risk phenotype.

## Supplementary material

10.1136/openhrt-2026-004514 online supplemental file 1

10.1136/openhrt-2026-004514 online supplemental file 2

10.1136/openhrt-2026-004514 online supplemental file 3

## Data Availability

Data are available upon reasonable request.
