# Supplementary material for: Association of exercise blood pressure relative to fitness with coronary artery disease
Source: Open Heart. 2026 Oct 21;13(2):e004514. doi: 10.1136/openhrt-2026-004514 (PMC13691892; doi:10.1136/openhrt-2026-004514)
Supplement: Supplementary file 1 [file openhrt-13-2-s001.pdf]

Supplementary Materials

Supplementary Figure 1, Relationship of CAD, inducible ischemia, and myocardial scar compared to exercise systolic BP relative to fitness

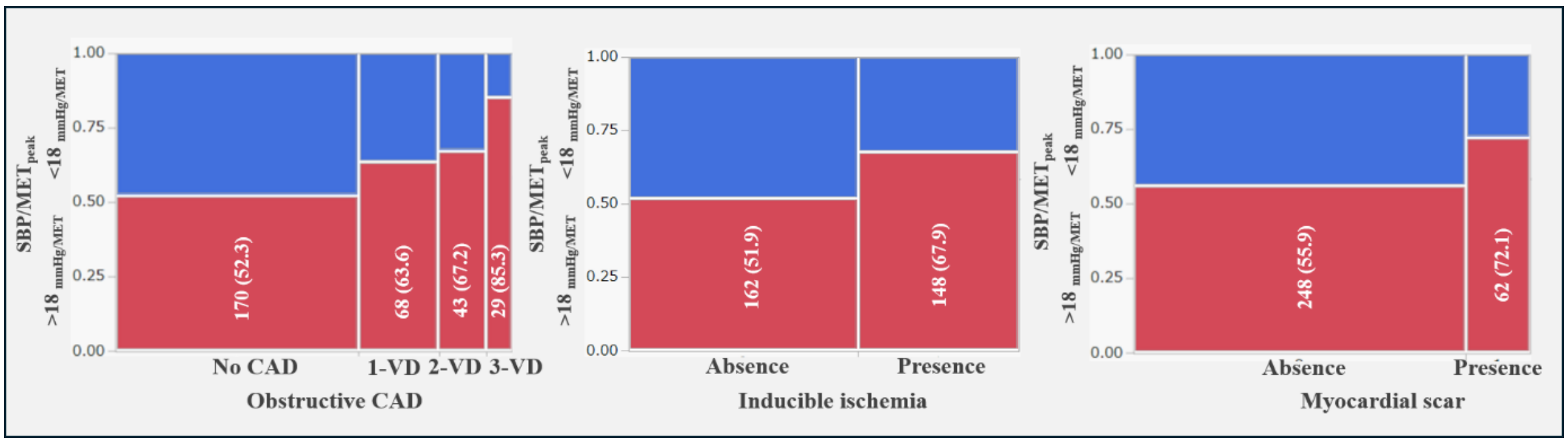

The prevalence of high SBP/MET_{peak} >18mmHg/MET was lowest in those without obstructive CAD and increased progressively with greater angiographic disease extent. Participants with myocardial scar or inducible ischemia had a significantly higher frequency of high SBP/MET_{peak} than those without scar.

SBP, systolic blood pressure; VD, vessel disease.

Supplementary Figure 2. Restricted cubic spline association between continuous SBP/METpeak and the primary outcome.

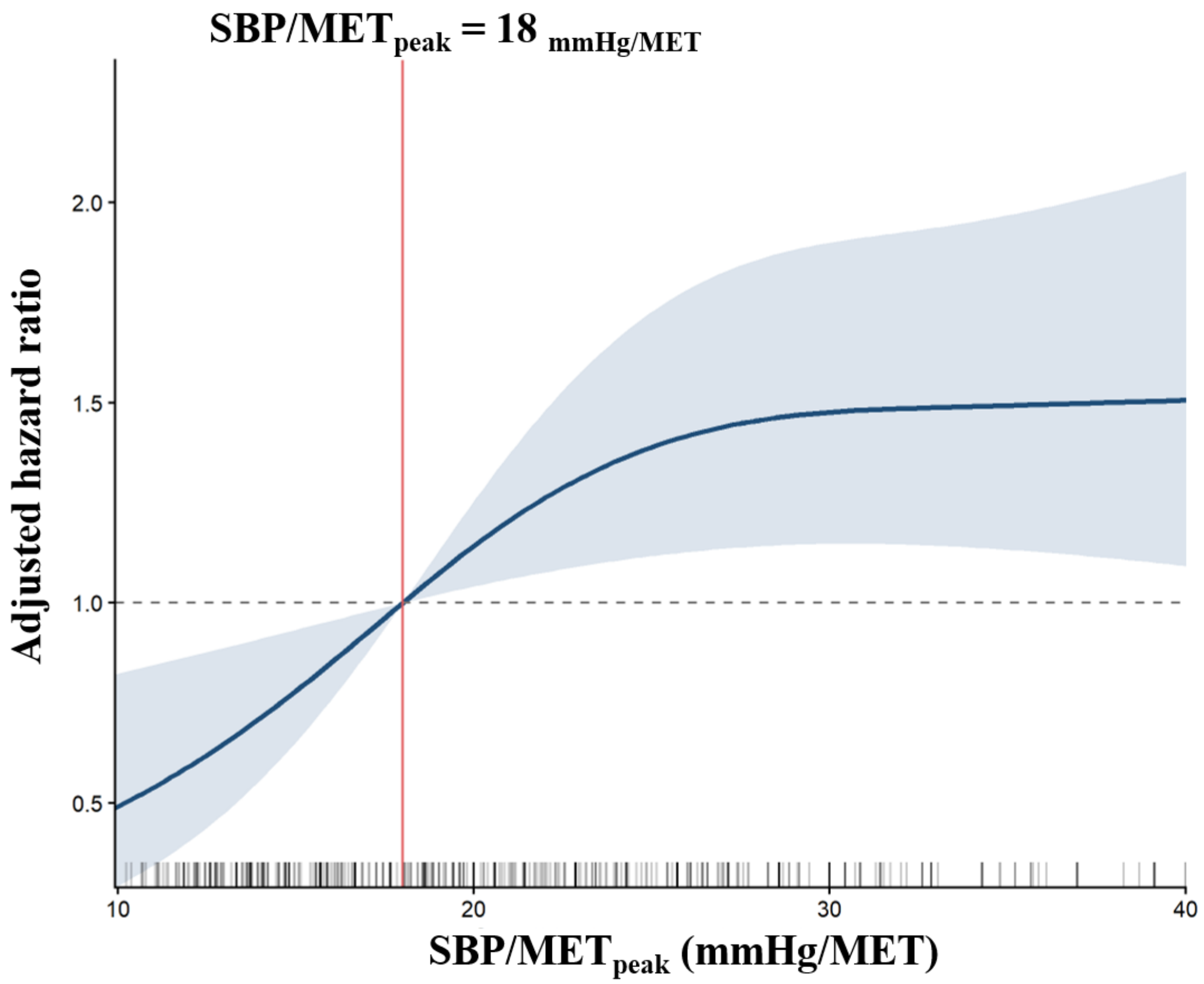

The solid curve represents the adjusted hazard ratio and the shaded band represents the 95% confidence interval. The model was adjusted for age, sex, obstructive coronary artery disease, inducible ischemia, and myocardial scar. Three knots were placed at the 10th, 50th, and 90th percentiles (12.6, 19.7, and 32.9 mmHg/MET), with 18 mmHg/MET as the reference. The overall association was significant (p=0.010), with evidence of nonlinearity (p=0.020).

Supplementary Table 1. Association of exercise systolic BP relative to fitness with clinical history of ACS in patients with myocardial infarction detected by LGE

	SBP/MET_{peak}		
	>18 mmHg/METs	<18 mmHg/METs	p value
With history of ACS	14 (58)	10 (42)	0.081
Without history ACS	48 (77)	14 (23)	

Abbreviation as in Table 1 and 2.

Supplementary Table 2. Univariable and Multivariable Cox hazard proportional analysis of exercise systolic BP relative to fitness for predicting secondary outcomes

	Univariable analysis		Multivariable analysis*	
**Predictor**	**HR (95% CI)**	**P value**	**HR (95%CI)**	**P value**
**All-cause death**				
SBP/MET_{peak}	1.03 (1.02–1.04)	<0.001	1.01 (1.00–1.03)	0.032
**Cardiovascular death**				
SBP/MET_{peak}	1.05 (1.02–1.06)	<0.001	1.03 (1.00–1.05)	0.011
**Non-cardiovascular death**				
SBP/MET_{peak}	1.03 (1.02–1.04)	<0.001	1.01 (0.99–1.02)	0.284
**Acute coronary syndrome**				
SBP/MET_{peak}	1.00 (0.96–1.04)	0.808	1.00 (0.96–1.03)	1.000

HRs (95% CIs) were expressed per 1-unit increase in SBP/MET_{peak}. HR, hazard ratio; CI, confidence intervals; other abbreviation as in Table 1 and 2.

Supplementary Table 3. Tests of the proportional-hazards assumption

Term	Chi-square	df	p value
SBP/METpeak	0.696	1	0.404
Age	1.361	1	0.243
Male	1.187	1	0.276
Obstructive CAD	2.225	1	0.136
Inducible ischemia on CMR	1.179	1	0.278
Myocardial scar on CMR	0.002	1	0.961
Global	6.254	6	0.395

The proportional-hazards assumption was evaluated using Schoenfeld residuals.

Supplementary Table 4. Time-dependent discrimination and cutoff performance of SBP/MET$_{peak}$

Time (years)	Cutoff (mmHg/MET)	Sensitivity	Specificity	Youden index
5	18.0	80.9%	42.0%	0.230
5	20.6	71.5%	54.0%	0.255
10	18.0	76.3%	42.8%	0.190
10	20.6	67.8%	55.4%	0.232
15	18.0	77.9%	47.0%	0.250
15	20.6	71.4%	60.8%	0.322

The 18.0-mmHg/MET threshold was previously proposed. The 20.6-mmHg/MET threshold was derived exploratorily by maximizing the 15-year time-dependent Youden index in the present cohort.

Supplementary Table 5. Comparison of component-based multivariable Cox models for the primary outcome

Model	Predictor	HR (95% CI)	P value	AICc	Global χ^2
1	SBP/MET_{peak}	1.02 (1.00-1.03)	0.012	1636	90.9
2	Peak SBP	1.00 (0.99-1.00)	0.145	1639	87.7
3	Peak METs	0.88 (0.82-0.94)	<0.001	1626	101.2
4	Peak SBP<br>Peak METs	1.00 (0.99-1.00)<br>0.88 (0.83-0.94)	0.321<br><0.001	1627	102.2

All models were adjusted for age, sex, obstructive coronary artery disease, inducible ischemia, and myocardial scar. Hazard ratios are per 1-unit increase. AICc, corrected Akaike information criterion; CI, confidence interval; METs, metabolic equivalents; SBP, systolic blood pressure.

Supplementary Table 6. Incremental prognostic performance after adding continuous SBP/MET_{peak}

Baseline model	LR χ^2	p	Harrell C base / extended	15-year AUC base / extended	p
Clinical + CAD	7.04	0.008	0.725 / 0.732	0.779 / 0.782	0.511
Clinical + CMR	7.16	0.007	0.727 / 0.733	0.780 / 0.783	0.564
Clinical + SPECT	7.80	0.005	0.734 / 0.741	0.785 / 0.788	0.575

The clinical model included age, sex, BMI, hypertension, diabetes, current smoking, ACE inhibitor use, beta-blocker use, and calcium-channel blocker use. Extended models added continuous SBP/MET_{peak}. AUC, time-dependent area under the curve; LR, likelihood ratio.
